# Suppression of Ghrelin Exacerbates HFCS-Induced Adiposity and Insulin Resistance

**DOI:** 10.3390/ijms18061302

**Published:** 2017-06-19

**Authors:** Xiaojun Ma, Ligen Lin, Jing Yue, Chia-Shan Wu, Cathy A. Guo, Ruitao Wang, Kai-Jiang Yu, Sridevi Devaraj, Peter Murano, Zheng Chen, Yuxiang Sun

**Affiliations:** 1 Department of Internal Medicine, Zhengzhou University, Zhengzhou 450052, China; xiaojun05312201@126.com; 2 Department of Pediatrics, Baylor College of Medicine, Houston, TX 77030, USA; ligenl@umac.mo (L.L.); jingyue8@hotmail.com (J.Y.); 3 State Key Laboratory of Quality Research in Chinese Medicine, University of Macau, Macau 999078, China; 4 Department of Reproductive Medicine, Huazhong Science and Technology University, Wuhan 430030, China; 5 Department of Nutrition and Food Science, Texas A&M University, College Station, TX 77843, USA; cjwu@tamu.edu (C.-S.W.); cathyguo13@gmail.com (C.A.G.); Peter.Murano@ag.tamu.edu (P.M.); 6 Department of Internal Medicine, Harbin Medical University, Harbin 150081, China; ruitaowang@126.com (R.W.); drkaijiang@163.com (K.-J.Y.); 7 Department of Pathology and Immunology, Baylor College of Medicine; Houston, TX 77030, USA; sxdevara@texaschildrens.org; 8 Department of Biochemistry and Molecular Biology, University of Texas Health Science Center, Houston, TX 77030, USA; Zheng.Chen.1@uth.tmc.edu

**Keywords:** ghrelin, HFCS, sucrose, fructose, glucose, adiposity, insulin resistance, adipose tissue inflammation

## Abstract

High fructose corn syrup (HFCS) is widely used as sweetener in processed foods and soft drinks in the United States, largely substituting sucrose (SUC). The orexigenic hormone ghrelin promotes obesity and insulin resistance; ghrelin responds differently to HFCS and SUC ingestion. Here we investigated the roles of ghrelin in HFCS- and SUC-induced adiposity and insulin resistance. To mimic soft drinks, 10-week-old male wild-type (WT) and ghrelin knockout (*Ghrelin^−/−^*) mice were subjected to ad lib. regular chow diet supplemented with either water (RD), 8% HFCS (HFCS), or 10% sucrose (SUC). We found that SUC-feeding induced more robust increases in body weight and body fat than HFCS-feeding. Comparing to SUC-fed mice, HFCS-fed mice showed lower body weight but higher circulating glucose and insulin levels. Interestingly, we also found that ghrelin deletion exacerbates HFCS-induced adiposity and inflammation in adipose tissues, as well as whole-body insulin resistance. Our findings suggest that HFCS and SUC have differential effects on lipid metabolism: while sucrose promotes obesogenesis, HFCS primarily enhances inflammation and insulin resistance, and ghrelin confers protective effects for these metabolic dysfunctions.

## 1. Introduction

Obesity has reached epidemic proportions in both developed and developing countries; concurrently, the incidences of metabolic syndrome and type 2 diabetes are increasing rapidly [1]. According to the World Health Organization, 39% of adults aged 18 and over were overweight in 2014; among them, 13% were obese [2]. It has been suggested that fructose-sweetened beverage consumption increases visceral adiposity and insulin resistance more severely than sucrose-sweetened beverage consumption [3]. Sucrose, a disaccharide consisting of a fructose and a glucose through a β-1,4-glycosidic bond, is commonly known as table sugar, which has been the major sweetener used in food globally for a long time [4]. However, since the 1970s, an alternative sweetener high fructose corn syrup (HFCS) has largely replaced sucrose. Due to its potent sweetness and low production cost, HFCS is now the major sweetener in the food industry in the US. HFCS is now ubiquitous in soft drinks and processed foods: HFCS-55 (55% fructose and 45% glucose) is most commonly used in beverages, while HFCS-42 (42% fructose and 58% glucose) is most commonly used in baked foods [5]. The replacement of sucrose with HFCS is considered to have a major role in the pathogenesis of obesity and diabetes [6]. Studies have linked increased consumption of drinks containing either HFCS or fructose to obesity and/or insulin resistance [3,6,7,8,9]. One study reported that postprandial triglycerides were elevated by consumption of all fructose-containing beverages (HFCS, sucrose or fructose alone) compared to the glucose-containing beverages [9], suggesting that fructose, regardless of its form, promotes obesity. However, other studies showed that there are no differences between calories from HFCS and sucrose, arguing “calories are calories” [9,10,11]. To date, the cause-and-effect relationship between HFCS and obesity remains controversial. In-depth studies of the effects of the two most common sweeteners of HFCS and sucrose on adiposity, inflammation and insulin sensitivity are urgently needed.

Ghrelin is the only known orexigenic peptide, which plays an important role in regulating obesity and its related disorders, such as insulin resistance and diabetes [12,13]. Interestingly, it has been reported that ghrelin also exerts regulatory effects independent of food intake [14,15,16,17]. Ghrelin stimulates the release of growth hormone (GH), food intake and obesity through its endogenous receptor, the growth hormone secretagogue receptor (GHS-R) [13,18]. Ghrelin stimulates osteoblast expansion and hepatic glucose production by mechanisms independent from GHS-R [19,20]. Ghrelin secretion is suppressed by intake of glucose, whereas this effect is attenuated after dietary fructose consumption [21,22]. Together, these observations suggest that ghrelin may have a unique role in HFCS-induced metabolic disturbances.

In this study, we assessed WT and ghrelin-ablated mice (*Ghrelin^−/−^*) under three different feeding conditions: (1) regular diet with plain drinking water (RD); (2) regular diet with 8% HFCS-55 in drinking water (HFCS); or (3) regular diet with 10% sucrose in drinking water (SUC). 8% HFCS and 10% sucrose were used to mimic soft drinks, and they were matched for similar total calories [23]. In the present study, HFCS-fed mice showed the most severe insulin resistance among three groups. The HFCS was detrimental to insulin sensitivity, irrespective of caloric intake or total body fat percentage. Moreover, ghrelin ablation further worsened HFCS-induced adiposity and insulin resistance. Taken together, the results indicate that HFCS consumption has negative effect on insulin sensitivity, and ghrelin exerts a protective role in HFCS-induced inflammation and insulin resistance.

## 2. Results

### 2.1. Effect of HFCS and SUC on Energy Metabolism

**Figure 1:**
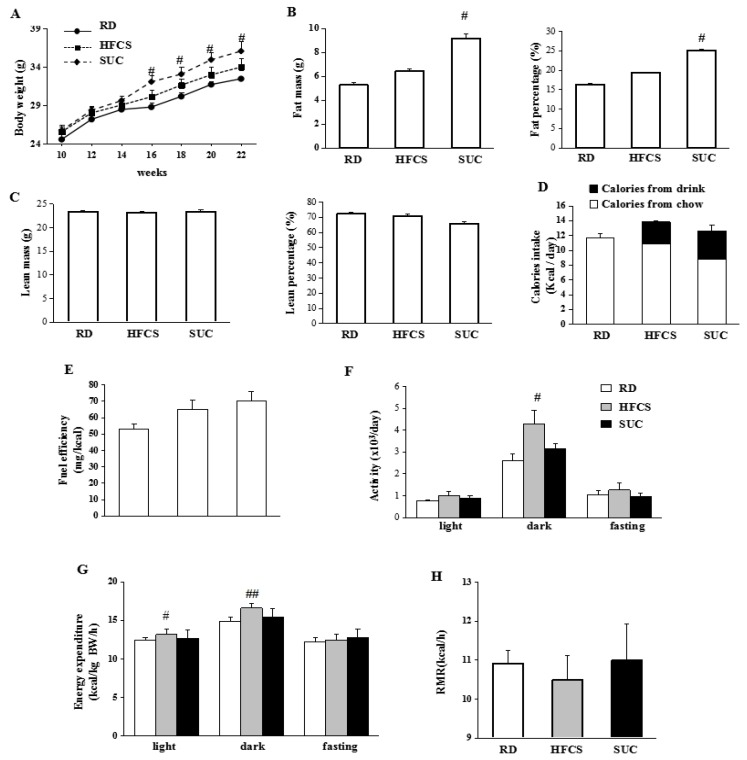
The metabolic parameters of wild-type (WT) mice under different diets. The WT mice were randomly divided into 1 of the 3 feeding regimens from 2 month old. Comprehensive Lab Animal Monitoring System (CLAMS) was performed after 3 month feeding: regular diet with drinking water (RD), regular chow with 8% high fructose corn syrup in the drinking water (HFCS), or regular chow with 10% sucrose in the drinking water (SUC). (**A**) Body weight of the mice with different feeding regimens; (**B**) Fat mass and fat percentage of 5 months old mice; (**C**) Lean mass and lean percentage of 5 months old mice; (**D**) Daily caloric intake (including calorie from both solid diet and drinks) of 4–5 months old mice; (**E**) Feeding efficiency of 5 months old mice; (**F**) Locomotor activity; (**G**) Energy expenditure (EE). (**H**) Resting metabolic rate (RMR). Each group contained 8–10 mice. #, *p* < 0.05, ##, *p* < 0.001, RD vs. HFCS or RD vs. SUC.

10-week-old WT mice were randomly divided into 3 groups, and received RD, HFCS or SUC for 12 weeks. Compared to RD group, the body weight of mice in SUC group was significantly higher from 16-week-old on, while the body weight of mice in HFCS group was only slightly higher (Figure 1A). Mice in HFCS group had similar fat and lean mass compared to RD group (Figure 1B,C), SUC-fed mice on the other hand had significantly higher fat mass and fat percentage than those of RD group, whereas the lean mass and lean percentage were comparable (Figure 1B,C). Total calorie intake was not significantly different between HFCS and SUC groups, while it was slightly lower in the RD group (Figure 1D). Fuel efficiency, defined as body weight gain/food intake, was slightly higher in the HFCS group compared to RD group, but lower than the SUC group (Figure 1E).

HFCS-fed mice showed higher locomotor activity during the dark period compared to RD and SUC groups, assessed by indirect calorimetry analysis (Figure 1F). Consistently, energy expenditure of HFCS-fed mice was elevated during both light and dark periods compared to RD- and SUC groups (Figure 1G). There were no statistical differences in the resting metabolic rate (RMR) among the 3 groups (Figure 1H).

Thus, while both HFCS and SUC showed a trend of increase in body weight and fat mass, only the effects observed in the SUC group were statistically significant. The greater fuel efficiency resulting from SUC-feeding likely contributed to the higher body weight and body fat in SUC-fed mice. On the other hand, the higher locomotor activity observed in HFCS-fed mice likely reduces the fuel efficiency.

### 2.2. Effect of HFCS and SUC on Insulin Sensitivity

**Figure 2:**
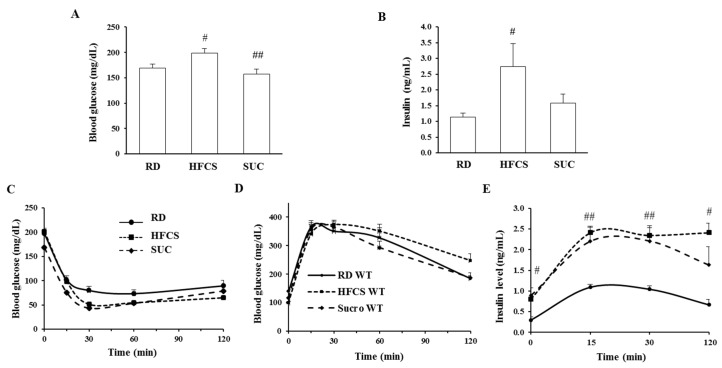
HFCS induces severe insulin resistance. Insulin tolerance test (ITT) and glucose tolerance test (GTT) were performed on 4-month-old mice. (**A**,**B**) Glucose and insulin levels after 6 h fasting; (**C**) Glucose levels during ITT; (**D**,**E**) Glucose and insulin levels during GTT. Each group contained 6–8 mice, #, *p* < 0.05, ##, *p* < 0.001, RD vs. HFCS or SUC.

To determine the effect of HFCS-and SUC-feeding on glucose homeostasis, blood glucose and insulin were measured after 10 weeks of HFCS or SUC feeding. The glucose level of HFCS-fed mice was significantly higher than those of RD- and SUC-fed mice, which was in line with the insulin levels (Figure 2A,B). During insulin tolerance tests (ITT), compared to the RD group, both SUC- and HFCS-feeding mice showed altered insulin responsiveness (Figure 2C). In glucose tolerance tests (GTT), HFCS- and SUC-feeding mice displayed similar glucose clearance rates when compared to RD group, but plasma insulin levels of both were significantly higher (Figure 2D,E). Consistently, HOMA-IR measurement showed that both HFCS- and SUC-feeding were associated with insulin resistance: RD (2.33 ± 0.74), SUC (6.36 ± 1.25) and HFCS (4.38 ± 0.40); *p* = 0.028, SUC vs. RD; *p* = 0.031, HFCS vs. RD. These data indicate that both HFCS- and SUC-fed mice were more insulin resistant than RD-fed mice.

### 2.3. Ablation of Ghrelin Exacerbates HFCS-Induced Fat Deposition

**Figure 3:**
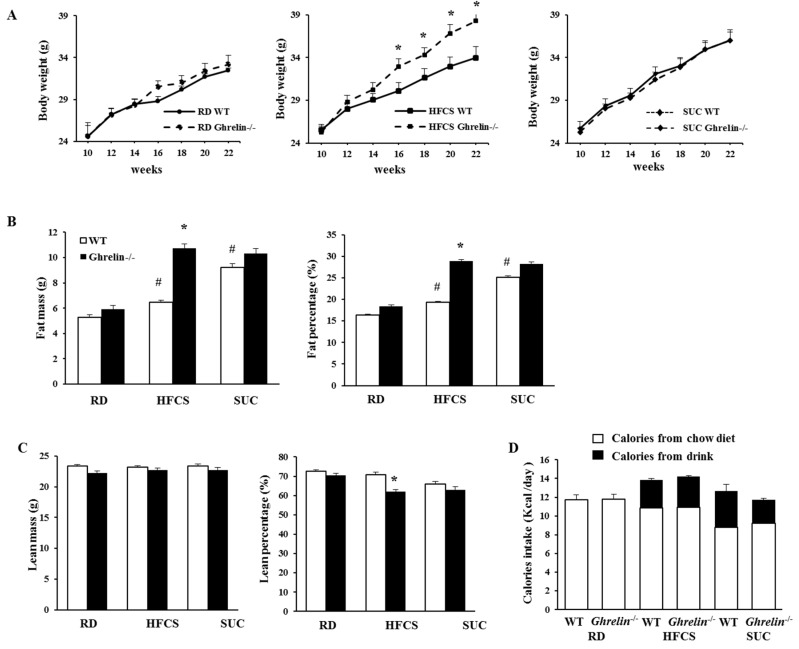
Ghrelin ablation exacerbates HFCS-induced adiposity. All three groups of mice were fed with RD, HFCS or SUC from 2 month old. (**A**) Body weight; (**B**,**C**) Weight and percentage of fat and lean mass of 5 months old mice; (**D**) Daily caloric intake (including calories from both solid diet and drink) of 4–5 months old mice. Each group contains 6–8 mice, * *p* < 0.05, *Ghrelin^−/−^* vs. WT mice. # *p* < 0.05, HFCS or SUC vs. RD.

Dietary fructose attenuates postprandial ghrelin suppression, and fructose and sucrose regulate ghrelin secretion differently [21]. Our previous study showed that total ghrelin level in mice was higher under HFCS feeding compared to RD feeding [24]. To assess whether ghrelin mediates the effects of HFCS and SUC, WT and *Ghrelin^−/−^* mice were given either SUC or HFCS from 10-weeks old of age. Surprisingly, *Ghrelin^−/−^* mice in HFCS group showed greater body weight gain, but not SUC-fed *Ghrelin^−/−^* mice (Figure 3A). Compared to WT mice, HFCS fed *Ghrelin^−/−^* mice had much higher body weight and fat content, while lean body mass was remained unchanged (Figure 3B,C). Our results showed that total energy intake was not changed in HFCS or SUC fed *Ghrelin^−/−^* mice (Figure 3D).

**Figure 4:**
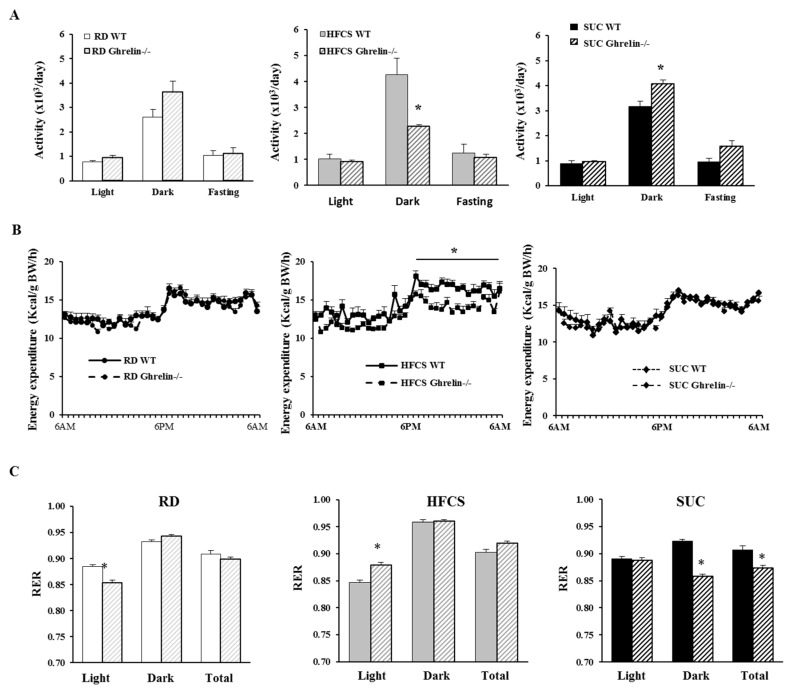
Ghrelin ablation decreases physical activity and total energy expenditure under HFCS feeding. CLAMS was performed on 5 months old mice. (**A**) Locomotor activity; (**B**) Energy expenditure; (**C**) Respiratory exchange ratio (RER). Each group contained 6–8 mice, * *p* < 0.05, WT vs. *Ghrelin^−/−^* mice.

Next, we carried out indirect calorimetry analysis. In HFCS group, *Ghrelin^−/−^* mice showed reduced physical activity during the dark cycle compared to WT mice; in SUC group, *Ghrelin^−/−^* mice exhibited increased physical activity during the dark period compared to WT mice (Figure 4A). Remarkably, HFCS feeding *Ghrelin^−/−^* mice showed reduced energy expenditure than that of HFCS fed WT mice, while there was no change between *Ghrelin^−/−^* and WT mice under SUC feeding (Figure 4B). We also analyzed respiratory exchange ratio (RER), an indicator of what type of fuel is being metabolized to supply energy for the body. Our data demonstrated that HFCS-fed *Ghrelin^−/−^* mice displayed a higher RER compared to SUC-fed *Ghrelin^−/−^* mice, which suggested that both fat and carbohydrate were used as fuel source for SUC-fed *Ghrelin^−/−^* mice, and carbohydrate was the primary fuel source for HFCS-fed *Ghrelin^−/−^* mice (Figure 4C). Collectively, the data indicate that ghrelin ablation exacerbates HFCS-induced adiposity, which was likely due to reduced physical activity.

### 2.4. Ghrelin Ablation Exacerbates HFCS-Induced Insulin Resistance

**Figure 5:**
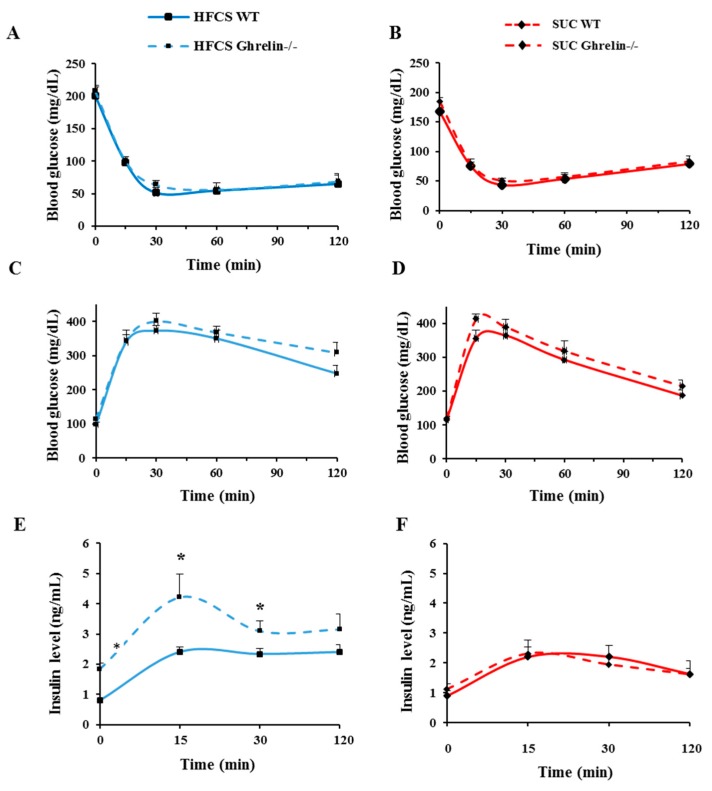
Ablation of ghrelin worsens HFCS-induced insulin resistance. (**A**,**B**) Glucose level during ITT of mice fed with HFCS or SUC; (**C**,**D**) Glucose level during GTT of mice fed with HFCS or SUC; (**E**,**F**) Insulin level during GTT of mice fed with HFCS or SUC. Each group contained 5 mice, * *p* < 0.05, WT vs. *Ghrelin^−/−^* mice under HFCS or SUC.

ITT and GTT were performed on 5-month-old WT and *Ghrelin^−/−^* mice to test the effect of ghrelin on insulin action with HFCS- and SUC-feeding regimen. During ITT, there was no significant difference in glucose levels between *Ghrelin^−/−^* and WT mice in either HFCS or SUC groups (Figure 5A,B). Similarly, during GTT, no difference in glucose levels was found under HFCS- or SUC condition between the two genotypes (Figure 5C,D); however, in HFCS group, plasma insulin levels in *Ghrelin^−/−^* mice were significantly increased compared to that of WT mice (Figure 5E); thus, ablation of ghrelin increased insulin resistance under HFCS-feeding. Plasma insulin levels remained unchanged between SUC-fed *Ghrelin^−/−^* mice and WT mice (Figure 5F).

### 2.5. Ghrelin Ablation Promotes Adipose Tissue Inflammation

**Figure 6:**
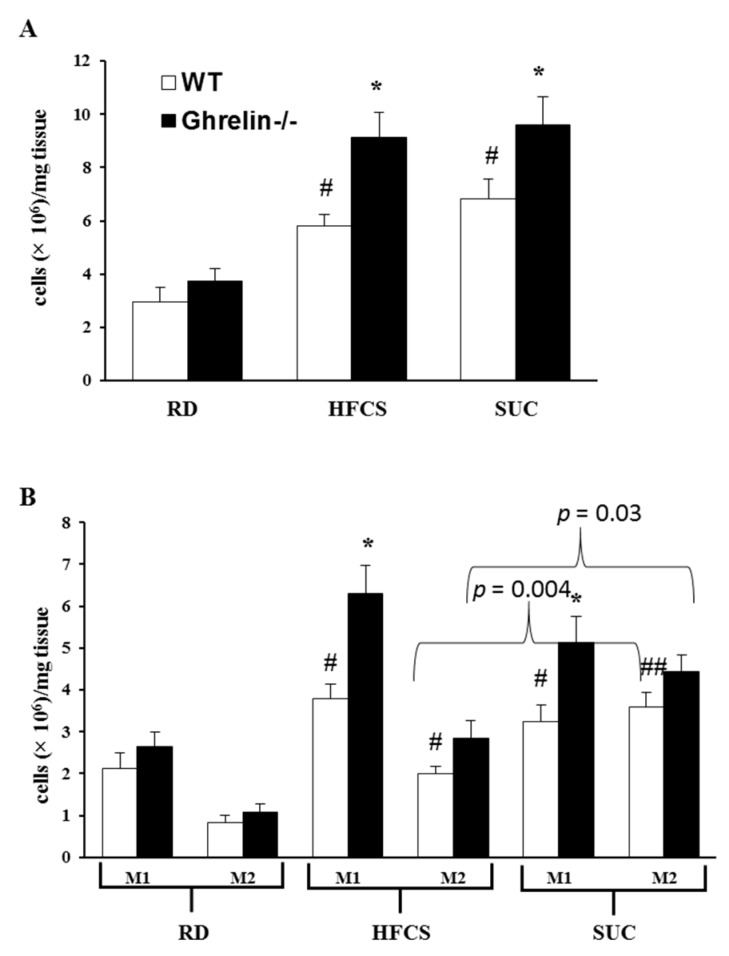
Effects of ghrelin deficiency on inflammation profile of adipose tissue macrophages. (**A**) Total macrophage contents in epididymal fat of the mice in three groups; (**B**) M1-like and M2-like macrophage content in epididymal fat of these three groups of mice. Each group contained 6 mice, * *p* < 0.05, WT vs. *Ghrelin^−/−^* mice; #, *p* < 0.05, ##, *p* < 0.001, RD vs. HFCS or SUC.

It has been established that adipose tissue inflammation can lead to insulin resistance; both macrophage infiltration and secretion of pro-inflammatory cytokines contribute to adipose inflammation [25,26,27,28]. We and others have reported that ghrelin is expressed in peritoneal macrophages and adipose tissue macrophages (ATMs) [24,29,30,31]. In the current study, we isolated ATMs from epididymal fat and carried out flow cytometry analysis to elucidate the effect of ghrelin on adipose inflammation. F4/80^+^ total ATMs were increased significantly in SUC- and HFCS fed mice compared to RD-fed mice (Figure 6A). Interestingly, *Ghrelin^−/−^* mice had higher total ATMs contents than that of WT mice under HFCS and SUC feeding, but not under RD feeding (Figure 6A). While pro-inflammatory F4/80^+^CD11c^+^ ATMs were similarly increased in SUC- and HFCS-fed mice, HFCS feeding mice showed less anti-inflammatory F4/80^+^CD11c^−^ATMs compared to SUC feeding mice, regardless of genotypes (Figure 6B). *Ghrelin^−/−^* mice showed increased F4/80^+^CD11c^+^ ATMs in both HFCS and SUC groups compared to RD group, while F4/80^+^CD11c^−^ ATMs showed a slight trend of increase under the same feeding condition (Figure 6B). These data suggest that HFCS is more pro-inflammatory than SUC; and HFCS-fed *Ghrelin^−/−^* mice were more severely insulin resistant than SUC-fed *Ghrelin^−/−^* mice.

## 3. Discussion

Our results showed that HFCS-feeding in mice slightly increased body weight, fat deposition and total calorie intake, while significantly elevated physical activity and energy expenditure. SUC-feeding, on the other hand, induced greater adiposity but did not markedly affect total food intake, physical activity, or energy expenditure. Consistent with the findings from the HFCS study in lean women, our data indicate that HFCS does not have significant effect on obesity (body fat) [11]. Dietary fructose and glucose have been shown to differentially affect lipid- and glucose- metabolism [32]. Fructose is implicated in glycemic control and insulin resistance. Long-term fructose feeding can induce compensatory hyperinsulinemia [26,27,28], whereas short-term fructose does not stimulate insulin secretion [33]. Our GTT and plasma insulin results support that both HFCS- and SUC- feedings promote systemic insulin resistance. HFCS- and SUC feeding had similar effects on glucose clearance compared to RD feeding; however, the insulin levels were higher in HFCS-fed *Ghrelin^−/−^* mice than SUC-fed *Ghrelin^−/−^* mice during GTT. In our study, 8% HFCS-55 was used, which is same as that used in Bocarsly’s rat study [23]. As shown in Figure 3D, about 3.1 kcal/day from HFCS solution was consumed regardless of the genotype, which is roughly equivalent to ~20% of daily energy intake. Another mouse study used diet enriched of fructose; around 35% of daily calories consumption was from fructose [34]. In a study of obese/over-weight humans, ~25% daily energy was from fructose [3]. In study of lean women, HFCS-sweetened beverage contributed 30% of daily energy [11]. Thus, the fructose dose in our study is lower than most animal and human studies; the overt phenotype we observed is not likely due to higher dose of fructose. In conclusion, our study supports that fructose is a more potent inducer of insulin resistance, and HFCS may elicit greater detrimental effects on insulin sensitivity than SUC.

A major finding in the current study is that ghrelin deficiency exacerbates the negative effects of HFCS on adiposity and insulin resistance. It is intriguing that HFCS elicited worse inflammatory effect than SUC only under ghrelin deficient condition. Several studies have shown that ghrelin has anti-inflammatory property [31,35,36]. Low plasma ghrelin is associated with insulin resistance [37,38]. We postulate that ghrelin deficiency predisposes mice to dietary insult, thus showing worse inflammation and insulin resistance. The compositions of HFCS (55% fructose and 45% glucose) and SUC (50% fructose and 50% glucose) are different. Fructose fails to stimulate insulin or leptin, which trigger feelings of “fullness” while eating [21]. Reduced secretion of insulin and leptin in response to fructose ingestion may cause a failure in communicating energy availability accurately. Postprandial ghrelin secretion is attenuated by both dietary fructose and glucose, but the suppressive effect is much less pronounced after fructose consumption than glucose [21]. The dysregulation of postprandial insulin and leptin under ghrelin deficient condition may be the underpinning cause of the differential phenotypes observed in HFCS- and SUC-fed ghrelin null mice. To fully elucidate whether fructose is the underpinning cause of ghrelin dependent metabolic dysfunctions, further studies are needed. Also in our study, we found that HFCS-fed *Ghrelin*^−/−^ mice have dramatically increased body weight and fat content, but had similar food intake. This suggests that ghrelin suppresses HFCS-induced adiposity, and this effect is independent from ghrelin’s orexigenic effect. Interestingly, ablation of ghrelin alters neither body weight nor body composition under SUC-feeding, which indicates that ghrelin is not involved in SUC-induced adiposity. Thus, ghrelin has differential lipogenic effects in response to HFCS- or SUC-feeding.

It has been reported in our previous study that ghrelin signaling plays an important role in the regulation of glucose-induced insulin secretion and insulin sensitivity [17]. Compared to RD-feeding, HFCS consumption induced higher total ghrelin levels, which suggested that HFCS had different regulating effect on ghrelin signaling compared to RD diets [24]. We previously reported that HFCS has negative effects on energy metabolism. HFCS induces robust insulin resistance; interestingly, GHS-R ablation improves insulin resistance induced by HFCS-feeding [24]. In contrast to effects seen with GHS-R ablation, the current study shows that ghrelin ablation worsens HFCS-induced insulin resistance. It is well known that ghrelin achieves its biological functions through both GHS-R dependent and independent manners in various tissues [20,39,40]. Differential effects of ghrelin ablation vs. ghrelin receptor ablation on body composition and energy homeostasis were reported in our previous study [39]. The current study further supports the notion that GHS- R ablation and ghrelin ablation exert differential effects on sugar-induced insulin resistance: ghrelin deficiency worsens insulin sensitivity whereas GHS-R deficiency improves insulin sensitivity.

The preproghrelin gene encodes 3 peptides: acylated ghrelin (active ghrelin), des-acyl ghrelin (DAG) and obestatin [41]. In *Ghrelin*^−/−^ mice, all three peptides are absent; in *Ghsr^−/−^* mice, only ghrelin’s actions are blocked while the actions of DAG and obestatin are intact. It is possible that other ghrelin related peptides might contribute to the differential phenotypes of HFCS-fed *Ghrelin*^−/−^ and *Ghsr^−/−^* mice. Previous study found that DAG has robust effects on adipose tissues [42], which may be an underpinning mechanism preferentially involved in HFCS-induced lipid metabolism. DAG may function as a crucial mediator for HFCS-induced adiposity; DAG-mediated lipid metabolism may be unaffected under RD- and SUC-feeding, but compromised under HFCS-feeding. Ghrelin is known to decrease spontaneous physical activity (SPA) [43]. Consistently, we observed that SPA of *Ghrelin*^−/−^ mice was increased under SUC-feeding. However, the SPA effect was abolished under HFCS feeding, and *Ghrelin*^−/−^ mice showed decreased SPA under HFCS feeding; this is in agreement with the obesity phenotype of HFCS-fed *Ghrelin*^−/−^ mice. The intriguing differential activity phenotypes of *Ghrelin*^−/−^ mice fed with HFCS and SUC suggest nutrient-dependent effects of ghrelin on SPA. 

In previous study, we reported that HFCS-feeding induced insulin resistance is primarily mediated by adipose inflammation and liver steatosis [24]. While GHS-R is readily detectable in macrophages [31,35,36], it is not expressed in the hepatocytes [44]. We thus concluded that HFCS-driven M1 macrophage polarization in white adipose tissue is likely to be the key underlying mechanism for HFCS-induced insulin resistance. Macrophage infiltration is a major pathophysiology that controls inflammation and metabolic dysfunctions in tissues [45]. In both human monocytes and T cells, ghrelin has shown an anti-inflammatory role [24,29,30,31]. In current study, while elevated pro-inflammatory macrophages were detected in epididymal fat of both HFCS- and SUC-fed mice, reduced anti-inflammatory macrophages were detected in HFCS-fed mice but not SUC-fed mice. These results indicate that more severe adipose inflammation is induced by HFCS compared to SUC, which is in line with the exacerbated insulin resistant state exhibited by HFCS-feeding. Furthermore, in flow cytometry analysis, HFCS increased ATMs containing both F4/80^+^CD11c^+^ and F4/80^+^CD11c^−^ types; at the same time, ghrelin ablation selectively increased pro-inflammatory F4/80^+^CD11c^+^ ATMs but not anti-inflammatory F4/80^+^CD11c^−^ ATMs. These data imply that ablation of ghrelin exacerbates adipose tissue inflammation induced by HFCS by increasing pro-inflammatory F4/80^+^CD11c^+^ macrophage infiltration, thus adversely affecting insulin action in adipose tissues.

Taken together, our data indicate that: (1) both HFCS and SUC can promote adipose tissues pro-inflammatory F4/80^+^CD11c^+^ macrophage infiltration, leading to adipose inflammation and insulin resistance; (2) HFCS and SUC differentially regulate lipogenesis and adipose inflammation, leading to different metabolic outcomes. (3) Ghrelin deficiency selectively worsens HFCS-induced, but not SUC–induced, adiposity and insulin resistance, leading to more severe metabolic dysfunctions. While our results are consistent with ghrelin having anti-inflammatory effects, they also reveal that GHS-R and ghrelin exerts different effects on HFCS-induced adiposity and insulin resistance. These findings underscore the complexity of ghrelin signaling pathway and the need to define the mechanistic roles of ghrelin and its receptor in diet-induced lipid metabolism and inflammation.

## 4. Materials and Methods

### 4.1. Animals

*Ghrelin^−/−^* mice were generated as previously described [46]. All mice in present study were backcrossed onto a pure C57BL/6J background. 10-week-old male WT and *Ghrelin^−/−^* mice were given ad libitum either regular diet (RD) of TD. 2920X (Harlan Teklad, Madison, WI, USA): 16% fat, 60% carbohydrates, 24% protein calories, energy consumption was calculated using the formula of 3.1 kcal × food intake (g); or the regular diet with 8% HFCS-55 (HFCS) used in rats [23] (Formula 55, *v*/*v* dissolved in drinking water, Nature’s Flavors, Orange, CA, USA), energy consumption was calculated using the formula of (3.1 kcal × food intake (g)) + (0.24 kcal × drink intake (g)); or regular diet with 10% sucrose (Domino Specialty Ingredients company, West Palm Beach, FL, USA) for 12 weeks; energy consumption was calculated using the formula of (3.1 kcal × food intake (g)) + (0.4 kcal × drink intake (g)). Isoflurane was used as anesthesia when the mice were sacrificed for tissue collection. We measured the body weight and food intake of mice every week, and calculated fuel efficiency by weekly body-weight gain (mg)/weekly energy consumption (kcal). Blood glucose levels were measured monthly by One-Touch Ultra glucometer (Lifescan, Milpitas, CA, USA) at the same time of the day. All experimental procedures used were approved by the Institutional Animal Care and Use Committee at Baylor College of Medicine (AN-2770, approved on 7 August 2014), and all methods were performed in accordance with the relevant guideline and regulations.

### 4.2. Body Composition Analysis

As previously described, Echo MRI-100 whole-body composition analyzer (Echo Medical Systems, Houston, TX, USA) was used to evaluate whole-body fat and lean mass of mice [17,39].

### 4.3. Metabolic Characterizations

As previously described, Oxymax open-circuit indirect calorimetry system (Columbus Instruments, Columbus, OH, USA) was used to measured metabolic parameters [17,39]. The study was carried out after housed in metabolic cages individually for one week. The entire test time was 72 h and the data collected from 24 to 72 h (stable state) were analyzed. Carbon dioxide production (VCO_2_) and Oxygen consumption (VO_2_) were recorded individually. The ratio of VCO_2_/VO_2_ was considered as respiratory exchange ratio (RER). The formula for energy expenditure (EE) calculation was as follows: (3.815 + 1.232 × VCO_2_/VO_2_) × VO_2_. The EE values were then normalized by body weight.

### 4.4. Glucose Tolerance Tests (GTT) and Insulin Tolerance Tests (ITT)

ITT and GTT assays were conducted as previously described [17,47,48]. Briefly, ITT was carried out after a 6 h fast before 10 a.m. The dose of human insulin used in the test was 1.0 U/kg body weight, and the time point of blood glucose measurements were taken place before the i.p. injection of insulin, and at 30, 60, 90 and 120 min after insulin injection. GTT was performed after overnight fast. The dose of glucose used in the test was 2.0 g/kg body weight, and blood glucose values were collected before glucose injection, and at 15, 30, 60 and 120 min after glucose injection. Insulin levels were analyzed at the time points of 0, 15, 30 and 120 min during GTT.

### 4.5. Stromal Vascular Fraction Isolation and Flow Cytometry Analysis

Stromal vascular fraction was isolated from epididymal fat as described previously [26,27]. Briefly, about 1 g fresh epididymal fat was weighted and cut into small pieces with scissors in digestion buffer (Krebs-Ringer bicarbonate buffer (KRB) supplemented with 1 mg/mL collagenase Type I (Worthington Chemicals, Lakewood, NJ, USA)). After incubation for 30 min in 37 °C shaking water bath, the tissue slurry was filtered through nylon mesh to remove undigested tissue. After centrifuged at 2200 rpm, the pellet of stromal vascular fraction (SVF) was re-suspended and washed with KRB twice. Then, about 1 × 10^6^ SVF cells were re-suspended in PBS (100 μL) and incubated with corresponding antibodies. The antibodies of PE anti-mouse F4/80 (eBioscience, San Diego, CA, USA), FITC anti-mouse CD11c (BD Bioscience, San Jose, CA, USA) and purified CD16/CD32 (BD Bioscience) were used to label macrophages. The remaining SVF cells were incubated with nonspecific IgG (BD Bioscience) to evaluate background fluorescence. The flow cytometry analysis was carried out on a FACScan (BD Biosciences).

### 4.6. Statistical Analysis

Repeated ANOVA and two-tailed Student’s *t*-test were recruited to determine statistical significance between genotypes or treatments. The results were represented as: mean ± S.E.M. Statistical significance is set to a minimum of *p* < 0.05.

## 5. Conclusions

The recruitment of pro-inflammatory macrophages in intra-abdominal fat is promoted by both HFCS and SUC, inducing adipose inflammation, which subsequently leads to deleterious effects on insulin sensitivity. Our studies demonstrate that HFCS exacerbates more severe metabolic damages than SUC, which suggests that the metabolic perturbations caused by HFCS are above and beyond the extra calories associated; thus, “calories are not calories”. Moreover, we found that ghrelin has differential responses to different type of sugar, selectively alleviates HFCS-induced metabolic dysfunctions. Immunometabolism is a novel research area to investigate the interaction between immunology and metabolism [49]. Our present data highlight a major role of HFCS in immunometabolism; it is not obesity-related per se, but rather adipose inflammation- and insulin resistance-related. Ablation of ghrelin worsens obesity, insulin resistance and adipose inflammation induced by HFCS, indicating that ghrelin signaling is a key mediator of HFCS-related immunometabolic outcomes. Ghrelin may have protective effects on HFCS-induced metabolic damages, representing a novel pathway linking nutrient-sensing signals with adipose inflammation and insulin resistance.

## Abbreviations

ATMs	Adipose tissue macrophages
DAG	Des-acyl ghrelin
GHS-R	Growth hormone secretagogue receptor
GTT	Glucose tolerance tests
HFCS	High fructose corn syrup
HOMA-IR	Homeostasis model assessment-insulin resistance
ITT	Insulin tolerance test
RMR	Resting metabolic rate
RD	Regular diet
SUC	Sucrose
WAT	White adipose tissue
